# Machine learning evaluation of immune infiltrate through digital tumour score allows prediction of survival outcome in a pooled analysis of three international stage III colon cancer cohorts

**DOI:** 10.1016/j.ebiom.2024.105207

**Published:** 2024-06-15

**Authors:** Julie Lecuelle, Caroline Truntzer, Debora Basile, Luigi Laghi, Luana Greco, Alis Ilie, David Rageot, Jean-François Emile, Fréderic Bibeau, Julien Taïeb, Valentin Derangere, Come Lepage, François Ghiringhelli

**Affiliations:** a Centre de Recherche INSERM LNC-UMR1231, Dijon, France; b Cancer Biology Transfer Platform, Centre Georges-François Leclerc, Dijon, France; c Genetic and Immunology Medical Institute, Dijon, France; d Department of Medical Oncology, San Giovanni di Dio Hospital, Crotone, Italy; e Department of Medicine and Surgery, University of Parma, Parma, Italy; f Molecular Gastroenterology Laboratory, IRCCS Humanitas Research Hospital, Rozzano, Milan, Italy; g Paris-Saclay University, Versailles SQY University (UVSQ), EA4340-BECCOH, Assistance Publique-Hôpitaux de Paris (AP-HP), Ambroise Paré Hospital, Smart Imaging, Service de Pathologie, Boulogne, France; h Service d'Anatomie et Cytologie Pathologiques, CHU Côte de Nacre, Normandie Université, Caen, France; i Department of Pathology, Besançon University Hospital, Besançon, France; j Institut du Cancer Paris Cancer Research for Personalized Medicine, Assistance Publique-Hôpitaux de Paris (AP-HP), Hôpital Européen Georges Pompidou, Paris, France; k Centre de Recherche des Cordeliers, Institut National de la Santé et de la Recherche Médicale (INSERM), Centre National de la Recherche Scientifique, Sorbonne Université, Université Sorbonne Paris Cité, Université de Paris, Paris, France; l Department of Gastroenterology and Digestive Oncology, Georges Pompidou European Hospital, AP-HP Centre, Université Paris Cité, Paris, France; m University of Burgundy Franche-Comté, Dijon, France; n Fédération Francophone de Cancérologie Digestive, Centre de Randomisation Gestion Analyse, EPICAD LNC 1231, Dijon, France; o Service d'Hépato-gastroentérologie et Oncologie digestive, CHU de Dijon, France; p Department of Medical Oncology, Centre Georges-François Leclerc, Dijon, France

**Keywords:** Tumour infiltrating lymphocytes, Machine learning, Colonic neoplasms, Biomarkers, Prognosis

## Abstract

**Background:**

T-cell immune infiltrates are robust prognostic variables in localised colon cancer. Evaluation of prognosis using artificial intelligence is an emerging field. We evaluated whether machine learning analysis improved prediction of patient outcome in comparison with analysis of T cell infiltrate only or in association with clinical variables.

**Methods:**

We used data from two phase III clinical trials (Prodige-13 and PETACC08) and one retrospective Italian cohort (HARMONY). Cohorts were split into training (*N* = *692*), internal validation (*N* = *297*) and external validation (*N* = *672*) sets. Tumour slides were stained with CD3mAb. CD3 Machine Learning (CD3ML) score was computed using graphical parameters within the tumour tiles obtained from CD3 slides. CD3 infiltrates in tumour core and invasive margin were automatically detected. Associations of CD3 infiltrates and CD3ML with 5-year Disease-Free Survival (DFS) were examined using univariate and multivariable survival models by Cox regression.

**Findings:**

CD3 density both in the invasive margin and the tumour core were significantly associated with DFS in the different sets. Similarly, CD3ML score was significantly associated with DFS in all sets. CD3 assessment did not provide added value on top of CD3ML assessment (Likelihood Ratio Test (LRT), *p* = *0.13*). In contrast, CD3ML improved prediction of DFS when combined with a clinical risk stage (LRT, *p* = *0.001*). Stratified by clinical risk score (High or Low), patients with low CD3ML score had better DFS.

**Interpretation:**

In all tested sets, machine learning analysis of tumour cells improved prediction of prognosis compared to clinical parameters. Adding tumour-infiltrating lymphocytes assessment did not improve prognostic determination.

**Funding:**

This research received no external funding.

**Research in context**

**Evidence before this study**

T-cell immune infiltrates are robust prognostic variables in localised colon cancer. Evaluation of prognosis using artificial intelligence is an emerging field.

**Added value of this study**

In two phase III clinical trials and one retrospective cohort that included 1661 patients with stage III colon cancer, the CD3 machine learning score was significantly associated with DFS and improved prediction of DFS when combined with known clinical biomarkers as risk stage.

**Implications of all the available evidence**

Machine learning analysis of tumour cells improved prediction of prognosis compared to clinical parameters.

## Introduction

Colorectal carcinoma (CRC) is the second most common cancer in Europe and the most common digestive tract cancer. In 2020 in Europe, the estimated number of new patients with CRC was 520,000, accounting for 8% of all cancer deaths (245,000 deaths) in Europe.^1^ Localised tumours are cured by surgery. However, for stage III CRC, three to six months of adjuvant chemotherapy with an oxaliplatin-based chemotherapy is recommended.^2^ Identifying the patients who are most likely to have disease recurrence and metastasis after surgical resection and adjuvant chemotherapy is a major unmet need.

Microsatellite instability (MSI) accounts for around 12% of patients with stage III CRC.^3^ The MSI phenotype is correlated with a high level of tumour-infiltrating lymphocytes (TILs) and also associated with better outcome, thus suggesting a role of TILs in predicting prognosis. The immune contexture, which is determined by the density, composition, functional state and organisation of the leukocyte infiltrate of the tumour, can yield information that is relevant to cancer prognosis or prediction of a treatment response because of the capacity of tumour infiltrating immune cells to shape cancer cell growth. Accordingly, presence of T lymphocytes inside the tumour bed is a surrogate marker of immunosurveillance, which controls tumour growth. CD3 is expressed by both CD8 and CD4 T cells, and high expression of CD3 is related to better prognosis in multiple cancer types.^4^, ^5^, ^6^ TILs can be analysed by haematoxylin and eosin (HE) staining, but also using immunohistochemistry. The concept of the “Immunoscore®”, previously proposed by Galon et al., studies CD3 and CD8 tumour infiltration in both the tumour core and invasive margin, and has been evaluated in various studies to define patient prognosis.^7^ This type of analysis is associated with a lower risk of tumour dissemination and improved survival in colon carcinoma (CC).^8^ Using a centralised method including patients with stage I–III CC, the Immunoscore® was shown to be capable of distinguishing three categories of patients, namely with high, intermediate and low scores, with different levels of recurrence-free survival.^9^ This concept was further validated in analysis of patients with stage III CRC included in the IDEA France GERCOR-Prodige study.^10^

Additionally, non-immune factors are also associated with outcomes in localised CRC. Sidedness, tumour molecular characteristics such as RAS status, mismatch repair status and consensus molecular subtypes, can also be used to better determine prognosis.^11^, ^12^, ^13^, ^14^ Artificial Intelligence (AI) could be used to analyse virtual microscopic images and determine, with good accuracy, the prognostic and tumour molecular characteristics.^15^^,^^16^ We previously reported that machine learning analysis of CD3 slides using tumour cell DGMate (DiGital tumour pArameTErs) and tumour stromal characteristic could improve determination of outcome in a phase III clinical trial of adjuvant chemotherapy in CRC.^17^

The primary objective of this study was therefore to determine the capacity of a machine learning score, namely the CD3ML Score which extracts prognostic features from tumour cells, and automatic TIL quantification to predict disease-free survival (DFS) in different international cohorts of stage III CRC. The predictive value of the digital score for DFS in comparison with clinical prognostic factors was also investigated.

## Methods

### Study population

Different cohorts were used in this study (Supplementary Figure S1a and b). All these patients underwent surgical ablation of localised CCR, without neoadjuvant treatment.

We used the PETACC08 cohort, a European, phase III trial that studied stage III CC adjuvant treatment with 12 cycles of FOLFOX-4 or a combination of cetuximab and FOLFOX-4 (NCT00265811).^18^ All *2559* patients were originally included between 22 December 2005 and 5 November 2009. Microsatellite stable (MSS) status and KRAS mutational status were determined as previously described.^14^^,^^19^ Enrolled patients had all provided written informed consent for translational research. Only a total of *1152* patients from PETACC08 were included in this study, due to slide unavailability from the local pathologist, or the absence of written informed consent for ancillary studies, or incomplete clinical data. Patients with slides without tumour were also excluded from the study.

Next, we selected *398* stage III CC from the Prodige-13 study (NCT00995202).^20^ This randomised, prospective, multicentre study was designed to investigate the impact of both intensive radiological monitoring *vs*. standard monitoring, and Carcinoembryonic Antigen (CEA) monitoring *vs.* no monitoring, since 2009. All use of clinical data and tissue specimens was in compliance with the Federation Francophone de Cancérologie Digestive (www.ffcd.fr) guidelines. Of the total *398* patients, only *322* were analysed in the present study due to slide unavailability.

We used an Italian cohort named HARMONY, comprising formalin-fixed, paraffin-embedded (FFPE) tumour specimens from *400* consecutive patients with stage III colorectal cancer between 1997 and 2006. These patients had undergone radical surgery at the Humanitas Clinical and Research Centre (Milan, Italy) from 1997 to 2006. Only *187* patients were analysed due to slide unavailability.

Comparison of clinical data from included vs excluded patients was performed in each data set (PETACC08, Prodige-13 and HARMONY). Only patients with stage III were included in this comparison. We observed a significant difference in histopathological grade for the PETACC08 cohort, because a part of the unanalysed slides were classified as “Undifferentiated”, and in tumour location for Prodige-13 and HARMONY cohorts due to the presence of rectal cancer in unanalysed patients (Supplementary Table S1, Supplementary Figure S1b).

### Ethics

The study was conducted according to the guidelines of the Declaration of Helsinki and Good Clinical Practice guidelines (ClinicalTrials.gov Identifiers: NCT00265811, NCT00995202).

Studies were approved by Comités de protection des personnes (CPP) Ile-de-france VI for PETACC08 and CCP EST1 for Prodige-13.

Samples from HARMONY cohort were obtained complying with protocols approved by the local Ethical Committee and Institutional Review Board at Humanitas Clinical and Research Center.

All participants provided written informed consent.

The manuscript is reported in accordance with the STROBE, REMARK and TRIPOD recommendations.^21^, ^22^, ^23^

### Training and validation cohorts

To develop and validate the different scores and models as prognostic variables, we created a training cohort, an internal validation cohort, and an external validation cohort (Supplementary Figure S1a). PETACC08 was divided in two sub cohorts, stained in two different centres: PETACC08-subgroup 1 (named sub1 hereafter) and PETACC08-subgroup 2 (sub2) (see below, CD3 Staining). PETACC08 sub1 cohort was split into two different cohorts by random sampling, placing 70% of the patients in the training cohort (*N* = *692*) and 30% in the internal validation cohort (*N* = *297*). The external validation cohort was composed of PETACC08 sub2, Prodige-13 and HARMONY cohorts (*N* = *672*) (Supplementary Table S2).

### CD3 staining

CD3 staining of *989* slides from the PETACC08 sub1 samples was carried out in the laboratory of J.F.E. Slides were stained as previously described,^24^ using Bond-Max Fr4.0 (Leica Biosystem) with CD3 primary antibodies (clone F7.2.38, Agilent, RRID: AB_1148907). Once counterstained and permanently mounted, slides were digitised with a Nanozoomer HT2.0 (Hamamatsu) at ×20 magnification to generate a whole slide imaging (WSI) file in ndpi format.

Slide staining for the Prodige-13 cohort and *163* slides of the PETACC08 sub2, was carried out using the Autostainer 48 (Agilent) and anti-CD3 primary antibody (clone F7.2.38, Agilent, RRID: AB_1148907). Once counterstained and permanently mounted, slides were digitalised with a Nanozoomer HT2.0 (Hamamatsu) at ×20 magnification to generate a WSI file in ndpi format.

Slide staining for the HARMONY cohort was performed using Bond-Max Fr4.0 (Leica Biosystem) with CD3 primary antibodies (1:50, clone F7238; Dako, RRID: AB_1148907). Once counterstained and permanently mounted, slides were digitised with a Nanozoomer HT2.0 (Hamamatsu) at ×20 magnification to generate a WSI file in ndpi format.

### CD3 detection and models

Once digitalised, we applied our ColoClass software, previously published in 2020.^17^ Briefly, the whole slide image was tiled with QuPath software (v3) and 124 graphical parameters were extracted from within each tile.^25^ A random forest model was estimated to classify any tile detected on the whole slide. Coloclass was finally able to automatically detect Tumour Core (TC) by collating tumour tiles when the surface was big enough, and automatically determine its Invasive Margin (IM) by a 300 μm surrounding border of the TC. The number of CD3 positive cells/mm^2^ was then calculated in these specific areas respectively called CD3-TC and CD3-IM. Each score was dichotomised into two groups (Low and High) using the median of each cohort (PETACC08 sub1 training and validation, PETACC08 sub2, Prodige-13 and HARMONY).

### CD3ML score construction

For each slide, the mean of graphical parameters on tumour tiles in TC and IM zones was computed, yielding 124 graphical parameters for each slide. Then a LASSO algorithm was estimated on the PETACC08 sub1 training cohort to select variables related to DFS using the glmnet R package.^26^ A CoxBoost algorithm was also used with almost the same results (R library CoxBoost).^27^

The CD3ML score is estimated as the linear predictor of the Cox model constructed in the training cohort with variables selected via the LASSO procedure. For each cohort (PETACC08 sub1 training and validation, PETACC08 sub2, Prodige-13 and HARMONY), CD3ML score was dichotomised into two groups (Low and High) using the best cut-off strategy based on survival information (maxstat R library^28^). In this way, the cut-off was cohort-dependent.

### Statistics

Quantitative variables are described as median and Interquartile Range (IQR), and qualitative variables as number and percentage. Patient characteristics were compared by cohort (training, internal validation and external validation) or by CD3 or CD3ML status using the Chi-2 or Fisher's exact test for qualitative variables, and the Wilcoxon rank sum test for continuous variables, as appropriate. Correlations between continuous variables were quantified using Pearson's correlation coefficient. *p*-values were adjusted using Benjamini-Hochberg FDR correction and adjusted *p*-values<0.05 were considered significant.

Disease-Free Survival (DFS) was computed as time from diagnosis of localised disease to recurrence or death and was evaluated at 60 months. Patients who were alive with no progression at 60 months were censored.

Survival analysis was performed using the survival R library. The prognostic value of the different variables was tested using univariate or multivariate Cox models for DFS when conditions of the model validity were applicable. Proportional hazards assumptions were tested based on Schoenfeld residuals. When the proportionality assumption was not verified, for multivariate models we fit an extended Cox model, with time dependent coefficients for relevant variables; the time varying coefficient was described with a parametric time function. Survival probabilities were estimated using the Kaplan–Meier method and survival curves were compared using the log-rank test when appropriate. When the proportional hazards assumption was not checked, the estimated restricted mean survival time (RMST) for DFS at 60 months was assessed to compare groups of interest (SurvRM2 R library).^29^ *P*-values less than or equal to 0*.*05 were considered statistically significant. Nested models were compared using the Likelihood Ratio Test (LRT) and the Area Under the Curve (AUC). AUC was estimated using different methods depending on whether the proportional hazards assumption was verified or not (respectively SurvivalROC or RiskSetROC R libraries).^30^^,^^31^ The predictive power of the different models were compared using AUC with 1000 x bootstrap replications and Wilcoxon rank sum tests.

Statistical analyses were performed using the R software (http://www.R-project.org/) and graphs were drawn using GraphPad Prism version 9.0.2.

### Roles of funders

This research received no external funding.

## Results

### Patient characteristics

The study includes *1661* patients overall, obtained from the two phase III clinical trials (PETACC08 and Prodige-13) and the retrospective Italian cohort, HARMONY. The PETACC08 sub1 cohort was split into a training and internal validation set and the other cohorts were pooled for the external validation set (Supplementary Figure S1a). Mean patient age was 63 (IQR = [55, 69]) years with a range of 23–94 years. Three hundred and twenty-two (19%) patients presented T4 and *562* (34%) patients presented N2 tumour status, yielding a total of *746* (45%) patients with high risk stage 3 and *915* (55%) patients with low risk stage 3.

Seven hundred and six (42%) patients had right-sided tumours, *955* (58%) had left-sided. One hundred and forty-four (9%) patients were MSI (Microsatellite Instability)/dMMR (deficient Mismatch Repair) and *505* (45%) were KRAS mutant (information available respectively for *1543* and *1111* patients). Patient characteristics, stratified by cohort, are shown in Table 1. No clinical difference was observed between the training and internal validation cohort. Patients in the external validation cohort were older than in the training or internal validation cohorts (Wilcoxon test, *p* < *0.001;* Supplementary Table S3).

**Table 1:** Clinical characteristics of the study population (All patients, Training, Internal validation and External validation cohorts).

Variables	All patients *N* = *1661*	Training cohort *N* = *692*	Internal validation cohort *N* = *297*	External validation cohort *N* = *672*	*p*-value	Adjusted *p*-value
Sex					*0.2*	*0.5*
Male	965 (58%)	416 (60%)	160 (54%)	389 (58%)		
Female	696 (42%)	276 (40%)	137 (46%)	283 (42%)		
Age	63 (55, 69)	61 (54, 67)	62 (54, 67)	65 (57, 73)	***<0.001***	***<0.001***
≤60	699 (42%)	341 (49%)	135 (45%)	223 (33%)	***<0.001***	***<0.001***
>60	962 (58%)	351 (51%)	162 (55%)	449 (67%)		
Histopathological grade					***0.007***	***0.033***
Well/Moderately differentiated	1400 (84%)	564 (82%)	247 (83%)	589 (88%)		
Poorly differentiated	261 (16%)	128 (18%)	50 (17%)	83 (12%)		
T status					*0.8*	*0.9*
1–3	1339 (81%)	553 (80%)	243 (82%)	543 (81%)		
4	322 (19%)	139 (20%)	54 (18%)	129 (19%)		
N status					*0.2*	*0.5*
1	1099 (66%)	441 (64%)	201 (68%)	457 (68%)		
2	562 (34%)	251 (36%)	96 (32%)	215 (32%)		
Risk stage					*0.2*	*0.5*
Low	915 (55%)	363 (52%)	174 (59%)	378 (56%)		
High	746 (45%)	329 (48%)	123 (41%)	294 (44%)		
M status						
0	969 (58%)	581 (84%)	256 (86%)	132 (20%)		
1	2 (0.1%)	2 (0.3%)	0 (0%)	0 (0%)		
x	690 (42%)	109 (15.7%)	41 (14%)	540 (80%)		
MSI or MMR status					*>0.9*	*>0.9*
MSS or pMMR	1399 (91%)	620 (91%)	259 (90%)	520 (91%)		
MSI or dMMR	144 (9%)	63 (9%)	28 (10%)	53 (9.2%)		
Unknown	118	9	10	99		
Sidedness					*0.4*	*0.7*
Left	955 (58%)	410 (59%)	163 (55%)	382 (57%)		
Right	706 (42%)	282 (41%)	134 (45%)	290 (43%)		
Lung metastasis	83 (7.2%)	53 (7.7%)	20 (6.7%)	10 (6.1%)	*0.7*	*0.9*
Unknown	509	0	0	509		
Liver metastasis	109 (9.5%)	71 (10%)	28 (9.4%)	10 (6.1%)	*0.3*	*0.6*
Unknown	509	0	0	509		
Bone metastasis	8 (0.7%)	6 (0.9%)	1 (0.3%)	1 (0.6%)	*0.9*	*>0.9*
Unknown	509	0	0	509		
Other metastasis	59 (5%)	35 (5.1%)	13 (4.4%)	11 (6.7%)	*0.5*	*0.7*
Unknown	509	0	0	509		
KRAS					*0.5*	*0.7*
Mutated	505 (45%)	321 (46%)	134 (45%)	50 (41%)		
Wild-Type	606 (55%)	371 (54%)	163 (55%)	72 (59%)		
Unknown	550	0	0	550		
BRAF					*0.4*	*0.7*
Mutated	122 (11%)	75 (11%)	37 (13%)	10 (8.3%)		
Wild-Type	959 (89%)	598 (89%)	250 (87%)	111 (92%)		
Unknown	580	19	10	551		

Comparisons were performed between training, internal validation and external validation cohorts.

*p*-values and adjusted *p-*values <0.05 are highlighted in bold.

MSI: Microsatellite Instability; MMR: Mismatch Repair; MSS: Microsatellite Stable; dMMR: deficient Mismatch Repair; pMMR: proficient Mismatch repair.

We analysed the association between clinical variables and CD3 TC, IM and CD3ML scores. Patients classified as low risk seemed to have more CD3 TC and IM (significant only for CD3 TC in the internal validation cohort) and had significantly lower CD3ML score (Supplementary Figure S2a). CD3 TC and IM scores appeared to be overexpressed in patients with right-sided tumours (Supplementary Figure S2b). The CD3ML score was not significantly associated with sidedness. Patients with MSI or dMMR status had significantly higher expression of CD3 TC, the same trend was observed for CD3 IM (significant only for the training cohort). Conversely, these patients had lower CD3ML score in the training and internal validation cohorts. In the external validation cohort, patients with MSI or dMMR status seemed to have higher CD3ML but the difference was not significant (Supplementary Figure S2c). Moreover, CD3 TC, IM and CD3ML scores were not associated with KRAS or BRAF mutation, except in the training cohort, where patients with BRAF mutations had higher CD3 TC score (Supplementary Figure S2d and e).

Then, we analysed the links between CD3 TC, IM and CD3ML scores. In the whole cohort, CD3 TC and IM scores were significantly correlated (Supplementary Figure S3a). CD3 TC score, and the CD3 TC and CD3 IM scores sum were significantly negatively correlated with CD3ML score. We tested whether scores were different in the training and validation cohorts. CD3 TC, CD3 IM and CD3ML scores were significantly different in the external validation cohort (Supplementary Figure S3b, c and d).

### Association of CD3 and CD3ML scores with DFS

**Fig. 1:**
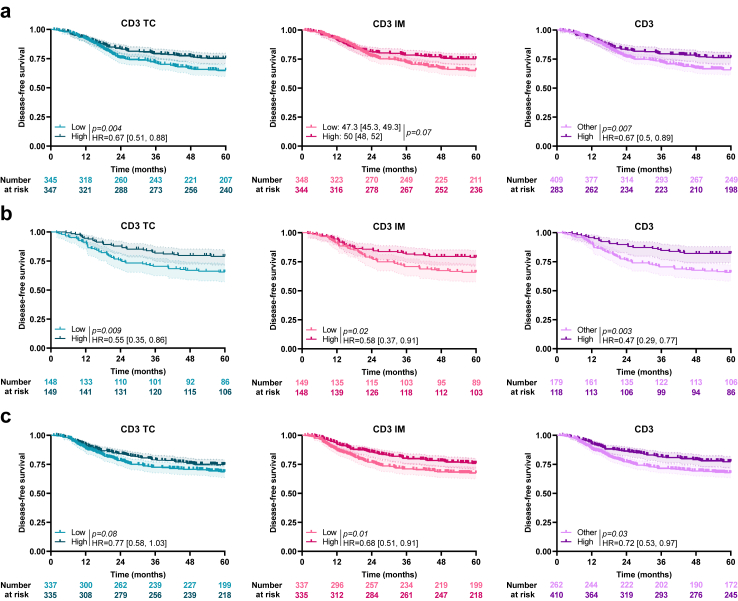
**Association between CD3 and survival**. Kaplan–Meier curves with patients stratified according to CD3 TC (blue), CD3 IM (pink) and combined CD3 (purple) status for disease-free survival in the a) training, b) internal validation and c) external validation cohort. Hazard ratio and log-rank test *p*-values were shown when proportional hazards assumption was verified, otherwise the RMST measure and *p*-value were presented. TC: Tumour Core; IM: Invasive Margin.

In the training cohort, using the median as a cut off, patients with high CD3 TC had significantly better survival than patients with low CD3 TC (HR = 0.67 [0.51, 0.88]; log-rank test, *p* = *0.004*). For CD3 IM, where condition of the Cox proportional model validity was not applicable, the estimated restricted mean survival time for DFS was used and at 60 months of follow-up, patients with high CD3 IM (using the median as a cut-off) seemed to have better survival than patients with low CD3 IM (47.3 [45.3, 49.3] months versus 50 [48, 52] months; RMST, *p* = *0.07*). A CD3 model was constructed using combined CD3 TC and CD3 IM status: patients with both high CD3 TC and high CD3 IM were considered as CD3^High^ and other patients (with either low CD3 TC and/or IM) as CD3^Other^. Patients classified CD3^High^ had better DFS than other patients (HR = 0.67 [0.5, 0.89]; log-rank test, *p* = *0.007*; Fig. 1a). Similar results were observed in the validation cohorts (internal and external) (Fig. 1b and c).

We tested the interactions between CD3 variable and clinical markers (Supplementary Table S4). In the training cohort, patients classified as CD3^High^ had more right-sided tumours and MSI/dMMR status than patients classified CD3^Other^. These observations were not significant in the validation cohorts.

**Fig. 2:**
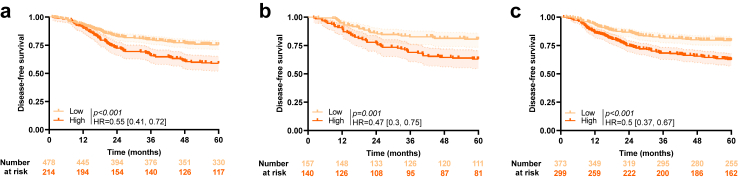
**Association between CD3ML score and survival**. Kaplan–Meier curves with patients stratified according to CD3ML status for disease-free survival in the a) training, b) internal validation and c) external validation cohort. Hazard ratio and log-rank test *p*-value were shown when proportional hazards assumption was verified otherwise the RMST measure and *p*-value were presented.

In the training cohort, using the optimal cut-off based on DFS, patients with low CD3ML score had significantly better survival than patients with high CD3ML score (HR = 0.55 [0.41, 0.72]; log rank test, *p* < *0.001*; Fig. 2a). The same observations were made for both the internal validation and external validation cohorts (respectively, HR = 0.47 [0.3, 0.75]; log-rank test, *p* = *0.001* and HR = 0.5 [0.37, 0.67]; log-rank test, *p* < *0.001*; Fig. 2b and c). When we divided the external validation cohort per sub-cohort, we observed similar significant results for Prodige-13 and PETACC08 sub2 cohorts, but results were not significant for the HARMONY cohort (data not shown). In all cohorts, patients classified CD3ML^Low^ were also mostly classified N1 and low risk, compared to patients classified CD3ML^High^ (Supplementary Table S5).

To go further, we pooled all cohorts and estimated a combined model with 4 modalities using CD3ML score and N status. In each of the 2 groups (N1 or N2), patients classified CD3ML^Low^ had better survival than patients classified CD3ML^High^ (N1: HR = 0.55 [0.43, 0.71]; log-rank test, *p* < *0.001;* N2: HR = 0.57 [0.44, 0.75]; log-rank test, *p* < *0.001*; Supplementary Figure S4).

**Fig. 3:**
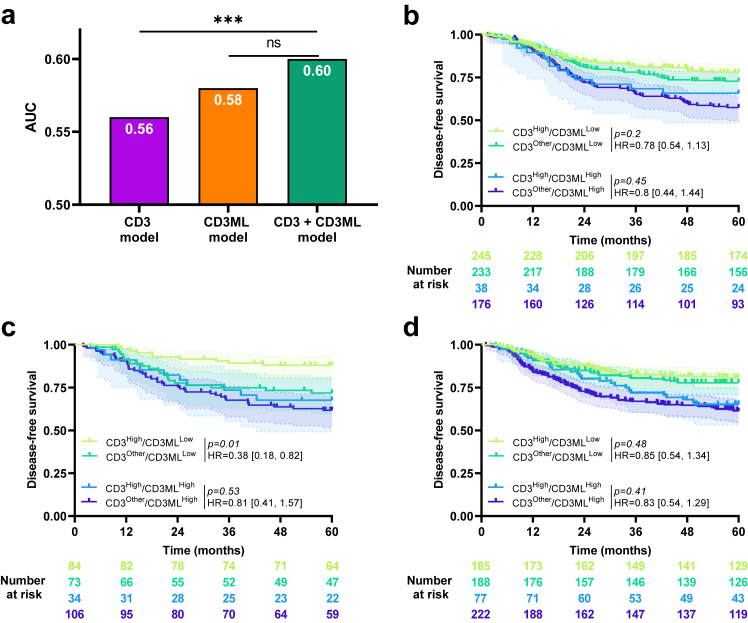
**Combination of CD3 and CD3ML**. a) Bar plots of time-dependent Area Under the Curve (AUC) for CD3, CD3ML and CD3 + CD3ML models for disease-free survival, ∗∗∗ Likelihood ratio test *p* < 0.001, ns: non-significant. Kaplan–Meier curves with patients stratified according to CD3 and CD3ML status for disease-free survival in the b) training, c) internal validation and d) external validation cohort. Hazard ratio and log-rank test *p*-value were presented when proportional hazards assumption was verified otherwise the RMST measure and *p*-value were presented.

We assessed the added value of the CD3ML and CD3 models in each cohort. In the training cohort, using time dependent area under the curve (AUC), we observed that the CD3 model did not add significant value on top of the CD3ML model (AUC = 0.58 for the CD3ML model and 0.6 for the CD3ML + CD3 model; LRT, *p* = *0.13*; Fig. 3a). By subgroup analysis, we observed no significant difference in DFS between CD3^High^ and CD3^Other^ patients in the CD3ML^Low^ group (HR = 0.78 [0.54, 1.13]; log-rank test, *p* = *0.2*) or in the CD3ML^High^ group (HR = 0.8 [0.44, 1.44]; log-rank test, *p* = *0.45*; Fig. 3b).

Findings were similar in both validation cohorts (Fig. 3c and d) except in the internal validation cohort which patients classified CD3^High^ had significant better DFS than patients classified CD3^Low^ in CD3ML^Low^ group (HR = 0.38 [0.18, 0.82]; log-rank test, *p* = *0.01).* Because the CD3 variable did not add any significant incremental value to the CD3ML variable, we pursued with the CD3ML-only model for further analysis.

CD3ML cut off is cohort dependent and we were not able to normalise CD3ML score. However, when slides were processed with the same technology (same CD3 staining and same digitalisation) we observed that cut-off could be validated across PETACC08 sub2 and Prodige-13 studies (data not shown).

### Combination of CD3ML and clinical variables

**Fig. 4:**
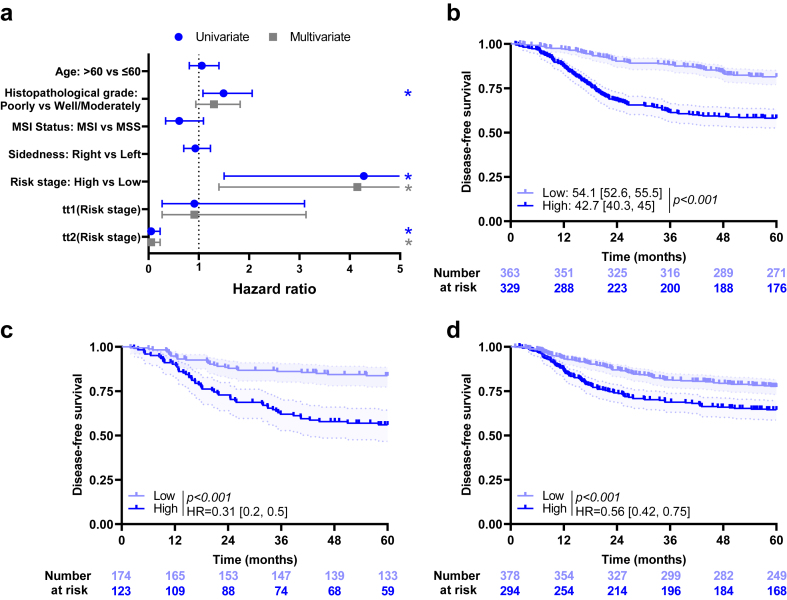
**Clinical variables and outcome**. a) Forest plots representing hazard ratios and confidence intervals for univariate (blue) and multivariate (grey). Cox models for disease-free survival estimated using clinical variables in the training cohort. ∗: Log-rank test *p*-value ≤0.05. Risk stage variable was estimated using time-dependent coefficient. Coefficients for tt1 (Risk stage) and tt2 (Risk stage) describe time varying effect of risk stage evaluated between 0 and 22 months, and 22 and 60 months, respectively. Kaplan–Meier curves with patients stratified according to the clinical model for disease-free survival in the b) training, c) internal validation and d) external validation cohort. Hazard ratio and log-rank test *p*-value were shown when proportional hazards assumption was verified otherwise the RMST measure and *p*-value were shown.

We performed Cox univariate analysis on the training cohort including only clinical variables available for at least 95% of all patients and for which proportional hazards assumption was verified (except M grade as all patients had the same modality, and T and N status as their information was included in risk stage). Patients with poorly differentiated tumour had lower DFS than the others (Fig. 4a). For sex and risk stage variables, condition of the Cox proportional model validity was not applicable. Using RMST measure, risk stage was significantly associated with DFS (RMST, *p* < *0.001).*

By multivariate Cox analysis including histological grade and risk stage using time-dependent coefficient, only risk stage remained significant (Fig. 4a).

A multivariate Cox model with risk stage and CD3ML score was estimated for each cohort, with risk stage estimated using time-dependent coefficient where necessary (Supplementary Figure S5). All variables were significantly and independently associated with DFS in the training and validation cohorts.

A clinical score was estimated using risk stage. Patients with low risk stage had better survival than patients with high risk stage (respectively 54.1 [52.6, 55.5] months and 42.7 [40.3, 45] months; RMST, *p* < *0.001*; Fig. 4b). Similar results were observed in the validation cohorts (Fig. 4c and d).

**Fig. 5:**
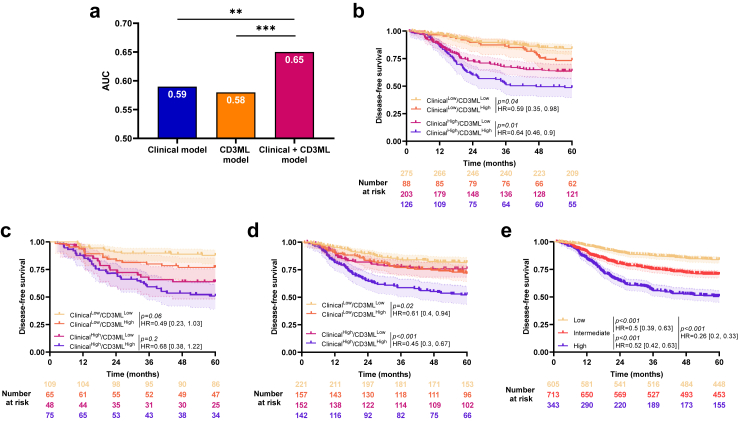
**Combination of clinical and CD3ML models**. a) Bar plots of time-dependent Area Under the Curve (AUC) for clinical, CD3ML and clinical + CD3ML models for disease-free survival, ∗∗*p* < 0.01 and ∗∗∗*p* < 0.001. Kaplan–Meier curves with patients stratified according to clinical and CD3ML status for disease-free survival in the b) training cohort, c) internal validation cohort and d) external validation cohort. e) Kaplan–Meier curves with patients stratified according to the combined, 3-modalities model for disease-free survival in all patients. Hazard ratio and log-rank test *p*-value were shown when proportional hazards assumption was verified otherwise the RMST measure and *p*-value were shown.

To test the capacity of the CD3ML variable to improve the predictive power of the clinical score, we generated a combined model. Comparison of models using the AUC and LRT showed that both variables improved the predictive power in the training cohort (AUC = 0.59 for the clinical model, 0.58 for the CD3ML model and 0.65 for the Clinical + CD3ML model; LRT, *p* < *0.001* between CD3ML model and Clinical + CD3ML model and LRT, *p* = *0.001* between Clinical and Clinical + CD3ML models; Fig. 5a). By subgroup analysis, CD3ML score was significantly associated with DFS, regardless of the clinical score. In both low and high clinical score groups, patients classified CD3ML^Low^ had better survival than patients classified CD3ML^High^ (HR = 0.59 [0.35, 0.98]; log-rank test, *p* = *0.04;* HR = 0.64 [0.46, 0.9]; log-rank test, *p* = *0.01*; Fig. 5b). Similar results were observed in both validation cohorts (Fig. 5c and d).

To go further, we pooled all cohorts and estimated a combined model with 3 modalities (High = Clinical^High^/CD3ML^High^, Intermediate = Clinical^High^/CD3ML^Low^ or Clinical^Low^/CD3ML^High^ and Low = Clinical^Low^/CD3ML^Low^). We observed that patients classified Low had better DFS than patients classified Intermediate (HR = 0.5 [0.39, 0.63]; log-rank test, *p* < *0.001*) or High (HR = 0.26 [0.2, 0.33]; log-rank test, *p* < *0.001*; Fig. 5e). Moreover, patients classified Intermediate had significantly better DFS than patients classed as High (HR = 0.52 [0.42, 0.63]; log-rank test, *p* < *0.001*).

Next, we analysed the predictive power of different variables using AUC with 1000 x bootstrap replications. When considering the whole cohort, CD3ML had a better AUC (median AUC = 0.58, IQR = [0.57, 0.6]) than risk stage (AUC = 0.56 [0.53, 0.6]; Wilcoxon test, *p* < *0.001*) and CD3 score (AUC = 0.55 [0.54, 0.56]; Wilcoxon test, *p* < *0.001*; Supplementary Figure S6a). In training cohort, risk stage had a better AUC than CD3 and CD3ML scores (risk stage: 0.6 [0.57, 0.62], CD3: 0.56 [0.54, 0.59] and CD3ML: 0.57 [0.56, 0.6]; Wilcoxon test, *p* < *0.001*, Supplementary Figure S6b). Similar results were observed in internal validation cohort (risk stage: 0.64 [0.62, 0.67], CD3: 0.59 [0.56, 0.59] and CD3ML: 0.59 [0.57, 0.62]; Wilcoxon test, *p* < *0.001*; Supplementary Figure S6c). In the external validation cohort, similarly at the whole cohort, CD3ML score (AUC = 0.59 [0.57, 0.61]) had better predictive capacity than risk stage (AUC = 0.57 [0.55, 0.59]; Wilcoxon test, *p* < *0.001*) and CD3 score (AUC = 0.54 [0.53, 0.56]; Wilcoxon test, *p* < *0.001*; Supplementary Figure S6d).

## Discussion

Various studies have demonstrated the prognostic role of CD3 immune infiltrate for improved assessment of patient prognosis in early stage CRC.^32^, ^33^, ^34^, ^35^, ^36^ Using a single CD3 slide, we were able to observe, in different datasets of patients treated for stage III CRC by surgery and adjuvant chemotherapy, that machine learning scoring of CD3 slides through our CD3ML model could predict DFS in each cohort. This information provides added value on top of clinical stage, and improves DFS prediction in patients with low or high clinical risk. Importantly, automatic counting of CD3 slides did not provide any added value on top of machine learning digital evaluation of CD3 slides, thus suggesting that machine learning digital assessment could be a simple way to overcome difficulties of TIL enumeration.

Artificial intelligence is a transformative innovation for pathology. Deep learning, a particular type of artificial intelligence, is a suitable technique to perform automatic delineation of tumour on a whole HE slide, and to predict the tumour type or some particular tumour genetic mutation.^37^ Previous studies have shown that deep learning could be used to develop a new histological classification to predict outcome in various tumour types, but also to unravel some particular morphological features related to outcome or response to therapy.^38^, ^39^, ^40^ Some retrospective studies have shown the capacity of this technique to predict survival in patients treated for localised colorectal cancer.^41^, ^42^, ^43^ Interestingly, such deep learning biomarkers provide added value on top of T and N stage to refine patient prognosis. Deep learning, by refining risk stratification, could improve clinical decision support for enhanced selection of adjuvant therapies. However, the implementation of such strategies in routine clinical practice remains challenging because of the computer time or graphics capacity required for such analysis. The models may also be difficult for physicians to interpret. Furthermore, external validation would be challenging because of the lack of reproducibility of HE staining across pathology laboratories, or even within the same laboratory, notably because of variations in fixation time.^44^^,^^45^

Alternative strategies to deep learning assessment of patient prognosis rely mainly on analysis of the immune contexture. To address this problem, the Immunoscore®, which counts CD3 and CD8 infiltrates in the tumour core and invasive margin, was previously proposed as a valuable strategy to predict patient outcome in early stage CRC. Results with the Immunoscore® were validated in various data sets and yielded results very similar to deep learning models.^9^^,^^46^ In a recent analysis of the IDEA France study, the prognostic value of the Immunoscore® was observed in patients with CRC stage III treated with oxaliplatin-based chemotherapy. A greater benefit of 6 months of adjuvant therapy was observed in patients with Immunoscore®-high, thus suggesting that this score could be valuable to stratify patient treatments.^33^ In the same context, Saberzadeh-Ardestani et al. demonstrated that TILs could be quantified based on HE slides, and there was an association in their study between high TIL infiltration and better DFS.^47^ They further observed that the prognostic features of TIL density were different depending on primary tumour sidedness and clinical risk group, suggesting that TILs should be interpreted in this context among stage III CRC.^47^ To go further, we recently reported that machine learning tumour assessment in addition to CD3 TIL analysis could be combined to improve prediction of patient prognosis in a single dataset.^17^

Our results show that despite automatic assessment, CD3 enumeration on a tumour slide may vary according to the dataset. This difficulty could arise from the fact that each cohort was stained in a different hospital using various technological platforms. To overcome this pitfall, the Immunoscore® requires central analysis. Further protocols remain proprietary, which strongly limits access to these types of analyses. To circumvent these limitations, a multistain deep learning pipeline was developed with the aim of predicting survival and treatment response in patients with CRC. This strategy demonstrated its efficacy for the prediction of relapse-free survival (RFS), but required laborious multiple chromogenic staining, which is expensive and not widely applicable in clinical routine.^48^

In this study, we used a simplified method. CD3ML is a score obtained from analysis of tumour core tissue using Qupath. QuPath was used to measure 124 parameters in each software segmented tile and the CD3ML score is the linear predictor of the Cox model with selected variables built on the PETACC08 cohort. The CD3ML score analyses tumour tissue structure as well as CD3 information. The correlation between CD3ML and CD3 scores, and the absence of any added value of CD3 counting on top of CD3ML demonstrates that CD3 enumeration could be outperformed by artificial intelligence evaluation of immune infiltrate without counting.

Based on recent studies demonstrating the value of immune checkpoint inhibitors in MSI tumours, we might suspect that CD3ML could be used to predict response to immunotherapy.^47^ Our results confirm this hypothesis. However, MSI tumours were treated with chemotherapy in this study, and further work is warranted to decipher whether CD3ML predicts response to immunotherapy.

The strengths of this study include the large number of patients coming from 2 prospective clinical trials with mature survival and well-characterised clinical data, and a large retrospective cohort from another country. This strategy helps us to generate training and validation sets, thereby strengthening our results. Limitations include the fact that our findings were not designed to determine an optimal universal cut off for CD3ML. The use of a LASSO algorithm and cut-off determination based on survival information represents a data-driven approach and may lead to overfitting. In this study, we did not validate the cut-off but, using internal and external validations, we were able to validate the predictive power of variables selected to compute the CD3ML score; in fact our score remained significant for each validation cohort. Variations in CD3ML or CD3 scores between cohorts suggest that for generalisation and prospective study, central analysis should be performed to avoid variations in the score related to technological variation in staining or digitalisation. Furthermore, all patients received adjuvant chemotherapy for 6 months, such that the predictive utility of TILs for chemotherapy outcomes could not be studied.

In conclusion, our study shows that patient tumours with low CD3ML had the best DFS in each dataset, and in patients with high or low risk. Prospective validation and determination of an optimal cut off are warranted, but these findings suggest that the CD3ML score should be interpreted in the context of tumour site for the purposes of estimating prognosis.

## Contributors

DB, LL, LG, AI, DR, JFE, FB, JT, CL, VD: data acquisition. JL, CT: data analysis. JL, CT, VD, FG: study design, data interpretation, manuscript and figures draft. JL, FG: Accessed and verified the data.

All authors read and approved the final version of the manuscript.

## Data sharing statement

Detailed extracted data on all included studies are available immediately following publication upon request to the corresponding author. Algorithms are available upon reasonable request to the corresponding author. Proposals should be directed by email to the corresponding author (FG), at the following email address: fghiringhelli@cgfl.fr.

## Declaration of interests

JT has received honoraria as a speaker and/or in an advisory role from AMGEN, Astellas, Astra Zeneca, BMS, Merck KGaA, MSD, Novartis, ONO pharmaceuticals, Pierre Fabre, Roche Genentech, Sanofi and Servier. CL has received honoraria as a speaker and/or in an advisory role from AMGEN, AAA-Novartis, Takeda, Deciphera, Pierre Fabre, Servier. FG's institution has received grants or contracts from Astra, consulting fees from Roche, AMGEN and MSD, honoraria as a speaker from Merck and support for attending meetings and/or travel from AMGEN. FB has received honoraria for lectures, speakers, presentations, manuscript writing or educational events from MSD, Pierre Fabre, Sanofi, BMS, Incyte, Servier and Astellas and support for attending meetings and/or travel from AMGEN, Pierre Fabre and MSD.
