# Supplementary material for: Machine learning evaluation of immune infiltrate through digital tumour score allows prediction of survival outcome in a pooled analysis of three international stage III colon cancer cohorts
Source: eBioMedicine. 2024 Jun 15;105:105207. doi: 10.1016/j.ebiom.2024.105207 (PMC11233898; doi:10.1016/j.ebiom.2024.105207)
Supplement: Supplementary Figures and Tables [file mmc1.pdf]

Supplementary materials

Table of contents

Supplementary Table 1: Comparison of clinical variables between included and excluded patients for each data set (PETACC08, Prodige-13 and HARMONY). Only patients with stage III colorectal cancer and clinical data were considered. 2

Supplementary Table 2: Comparison of clinical variables between external validation cohorts 3

Supplementary Table 3: Pairwise comparisons of clinical variables between cohorts. 4

Supplementary Table 4: Comparisons of clinical variables according to CD3 status (High vs Other) in each cohort 5

Supplementary Table 5: Comparisons of clinical variables according to CD3ML status in each cohort. 6

Supplementary Figure 1: Flowcharts of study 7

Supplementary Figure 2: Clinical variables and digital scores 8

Supplementary Figure 3: Association between CD3 and CD3ML scores 9

Supplementary Figure 4: Association between CD3ML score and N status in all cohort. 10

Supplementary Figure 5: Association between risk stage and CD3ML score in each cohort 11

Supplementary Figure 6: Predictive power of clinical and digital variables 12

Supplementary Table 1: Comparison of clinical variables between included and excluded patients for each data set (PETACC08, Prodige-13 and HARMONY). Only patients with stage III colorectal cancer and clinical data were considered.

Variables	PETACC08				Prodige-13				HARMONY			
	Excluded patients N=39	***Included patients N= 1.152***	**P-value**	**Adjusted p-value**	***Excluded patients N=289***	***Included patients N=322***	**P-value**	**Adjusted p-value**	***Excluded patients N=212***	***Included patients N=187***	**P-value**	**Adjusted p-value**
Sex			0.6	0.7			0.4	0.5			0.9	0.9
Male	21 (54%)	667 (58%)			175 (61%)	183 (57%)			129 (61%)	115 (61%)		
Female	18 (46%)	485 (42%)			114 (39%)	139 (43%)			83 (39%)	72 (39%)		
Age	60 (52, 67)	61 (54, 67)	0.6	0.7	66 (59, 73)	67 (60, 76)	0.052	0.2	69 (61, 77)	67 (60, 76)	0.3	0.5
≤60	20 (51%)	568 (49%)	0.8	0.9	83 (29%)	82 (25%)	0.4	0.5	49 (23%)	49 (26%)	0.5	0.7
>60	19 (49%)	584 (51%)			206 (71%)	240 (75%)			163 (77%)	138 (74%)		
Histopathological grade			**<0.001**	**<0.001**			0.5	0.5			0.15	0.5
Well/Moderately differentiated	16 (59%)	945 (82%)			273 (96%)	312 (97%)			147 (70%)	143 (76%)		
Poorly differentiated	5 (19%)	207 (18%)			12 (4.2%)	10 (3.1%)			63 (30%)	44 (24%)		
Undifferentiated	6 (22%)	0 (0%)			0	0			0	0		
Unknown	12	0			4	0			2	0		
T status			0.3	0.5			0.4	0.5			0.2	0.5
1-3	28 (74%)	927 (80%)			230 (82%)	255 (79%)			187 (89%)	157 (84%)		
4	10 (26%)	225 (20%)			51 (18%)	67 (21%)			24 (11%)	30 (16%)		
Unknown	1	0			8	0			1	0		
N status			0.2	0.4			0.3	0.5			0.2	0.5
0	0	0			2 (0.7%)	0 (0%)			0	0		
1	21 (54%)	744 (65%)			211 (73%)	228 (71%)			131 (62%)	127 (68%)		
2	18 (46%)	408 (35%)			76 (26%)	94 (29%)			81 (38%)	60 (32%)		
Risk Stage			0.11	0.3			0.2	0.5			0.6	0.7
Low	16 (41%)	622 (54%)			175 (62%)	185 (57%)			117 (55%)	108 (58%)		
High	23 (59%)	530 (46%)			106 (38%)	137 (43%)			94 (45%)	79 (42%)		
Unknown					8	0			1	0		
M status			0.12	0.3								
0	32 (82%)	969 (84%)			0	0						
1	1 (2.6%)	2 (0.2%)			0	0						
x	6 (15%)	181 (16%)			0	0						
MSI or MMR status			0.082	0.3			0.5	0.5			0.5	0.7
MSS or pMMR	32 (82%)	1,028 (91%)			21 (84%)	2 (67%)			21 (10%)	22 (12%)		
MSI or dMMR	7 (18%)	101 (8.9%)			4 (16%)	1 (33%)			189 (90%)	161 (88%)		
Unknown	0	23			264	319			2	4		
Sidedness			0.12	0.3			**<0.001**	**<0.001**			**<0.001**	**<0.001**
Left	18 (46%)	676 (59%)			121 (42%)	171 (53%)			75 (35%)	108 (58%)		
Rectum	0	0			89 (31%)	0 (0%)			64 (30%)	0 (0%)		
Right	21 (54%)	476 (41%)			79 (27%)	151 (47%)			73 (34%)	79 (42%)		
Lung metastasis	1 (2.6%)	83 (7.2%)	0.5	0.7	0	0			0	0		
Unknown	0	0			289	322			212	187		
Liver metastasis	5 (13%)	109 (9.5%)	0.4	0.7	0	0			0	0		
Unknown	0	0			289	322			212	187		
Bone metastasis	0 (0%)	8 (0.7%)	>0.9	>0.9	0	0			0	0		
Unknown	0	0			289	322			212	187		
Other metastasis	5 (13%)	59 (5.1%)	0.053	0.3	0	0			0	0		
Unknown	0	0			289	322			212	187		
KRAS			0.6	0.7								
Mutated	15 (41%)	505 (45%)			0	0			0	0		
Wild-Type	22 (59%)	606 (55%)			0	0			0	0		
Unknown	2	41			289	322			212	187		
BRAF			0.3	0.5	0	0						
Mutated	6 (16%)	122 (11%)			0	0			0	0		
Wild-Type	31 (84%)	959 (89%)			0	0			0	0		
Unknown	2	71			289	322			212	187		

MSI: Microsatellite Instability; MMR: Mismatch Repair; MSS: Microsatellite Stable; dMMR: deficient Mismatch Repair; pMMR: proficient Mismatch repair

Supplementary Table 2: Comparison of clinical variables between external validation cohorts

Variables	External validation cohort *N = 672*	Prodige-13 *N = 322*	PETACC08 sub2 *N = 163*	HARMONY *N = 187*	P-value	Adjusted p-value
Sex					*0.5*	*0.7*
Male	389 (58%)	183 (57%)	91 (56%)	115 (61%)		
Female	283 (42%)	139 (43%)	72 (44%)	72 (39%)		
Age	65 (57, 73)	67 (60, 76)	59 (53, 65)	67 (60, 76)	***<0.001***	***<0.001***
≤60	223 (33%)	82 (25%)	92 (56%)	49 (26%)	***<0.001***	***<0.001***
>60	449 (67%)	240 (75%)	71 (44%)	138 (74%)		
Histopathological grade					***<0.001***	***<0.001***
Well/Moderately differentiated	589 (88%)	312 (97%)	134 (82%)	143 (76%)		
Poorly differentiated	83 (12%)	10 (3.1%)	29 (18%)	44 (24%)		
T status					*0.4*	*0.7*
1-3	543 (81%)	255 (79%)	131 (80%)	157 (84%)		
4	129 (19%)	67 (21%)	32 (20%)	30 (16%)		
N status					*0.2*	*0.4*
1	457 (68%)	228 (71%)	102 (63%)	127 (68%)		
2	215 (32%)	94 (29%)	61 (37%)	60 (32%)		
Risk Stage					*0.5*	*0.7*
Low	378 (56%)	185 (57%)	85 (52%)	108 (58%)		
High	294 (44%)	137 (43%)	78 (48%)	79 (42%)		
M status					***<0.001***	***<0.001***
0	132 (20%)	0 (0%)	132 (81%)	0 (0%)		
1	0 (0%)	0 (0%)	0 (0%)	0 (0%)		
x	540 (80%)	322 (100%)	31 (19%)	187 (100%)		
MSI or MMR status					*0.2*	*0.4*
MSS or pMMR	520 (91%)	210 (91%)	149 (94%)	161 (88%)		
MSI or dMMR	53 (9.2%)	21 (9.1%)	10 (6.3%)	22 (12%)		
Unknown	99	91	4	4		
Sidedness					*0.1*	*0.3*
Left	382 (57%)	171 (53%)	103 (63%)	108 (58%)		
Right	290 (43%)	151 (47%)	60 (37%)	79 (42%)		
Lung metastasis	10 (6.1%)	0 (0%)	10 (6.1%)	0	*>0.9*	*>0.9*
Unknown	509	322	0	187		
Liver metastasis	10 (6.1%)	0 (0%)	10 (6.1%)	0	*>0.9*	*>0.9*
Unknown	509	322	0	187		
Bones metastasis	1 (0.6%)	0 (0%)	1 (0.6%)	0	*>0.9*	*>0.9*
Unknown	509	322	0	187		
Other metastasis	11 (6.7%)	0 (0%)	11 (6.7%)	0	*>0.9*	*>0.9*
Unknown	509	322	0	187		
KRAS						
Mutated	50 (41%)	0 (0%)	50 (41%)	0		
Wild-Type	72 (59%)	0 (0%)	72 (59%)	0		
Unknown	550	322	41	187		
BRAF					*>0.9*	*>0.9*
Mutated	10 (8.3%)	0 (0%)	10 (8.3%)	0		
Wild-Type	111 (92%)	0 (0%)	111 (92%)	0		
Unknown	551	322	42	187		

MSI: Microsatellite Instability; MMR: Mismatch Repair; MSS: Microsatellite Stable; dMMR: deficient Mismatch Repair; pMMR: proficient Mismatch repair

Supplementary Table 3: Pairwise comparisons of clinical variables between cohorts.

Variables	Training cohort *N = 692*	Internal validation cohort *N = 297*	P-value	Adjusted p-value	Training cohort *N = 692*	External validation cohort *N = 672*	P-value	Adjusted p-value	Internal validation cohort *N = 297*	External validation cohort *N = 672*	P-value	Adjusted p-value
Sex			*0.079*	*0.7*			*0.4*	*0.6*			*0.3*	*0.6*
Male	416 (60%)	160 (54%)			416 (60%)	389 (58%)			160 (54%)	389 (58%)		
Female	276 (40%)	137 (46%)			276 (40%)	283 (42%)			137 (46%)	283 (42%)		
Age	61 (54, 67)	62 (54, 67)	*0.3*	*0.8*	61 (54, 67)	65 (57, 73)	*<0.001*	***<0.001***	62 (54, 67)	65 (57, 73)	*<0.001*	***<0.001***
≤60	341 (49%)	135 (45%)	*0.3*	*0.8*	341 (49%)	223 (33%)	*<0.001*	***<0.001***	135 (45%)	223 (33%)	*<0.001*	***0.002***
>60	351 (51%)	162 (55%)			351 (51%)	449 (67%)			162 (55%)	449 (67%)		
Histopathological grade			*0.6*	*0.8*			*0.002*	***0.008***			*0.068*	*0.3*
Well/Moderately differentiated	564 (82%)	247 (83%)			564 (82%)	589 (88%)			247 (83%)	589 (88%)		
Poorly differentiated	128 (18%)	50 (17%)			128 (18%)	83 (12%)			50 (17%)	83 (12%)		
T status			*0.5*	*0.8*			*0.7*	*0.8*			*0.8*	*>0.9*
1-3	553 (80%)	243 (82%)			553 (80%)	543 (81%)			243 (82%)	543 (81%)		
4	139 (20%)	54 (18%)			139 (20%)	129 (19%)			54 (18%)	129 (19%)		
N status			*0.2*	*0.8*			*0.1*	*0.3*			*>0.9*	*>0.9*
1	441 (64%)	201 (68%)			441 (64%)	457 (68%)			201 (68%)	457 (68%)		
2	251 (36%)	96 (32%)			251 (36%)	215 (32%)			96 (32%)	215 (32%)		
Risk Stage			*0.082*	*0.7*			*0.2*	*0.4*			*0.5*	*0.8*
Low	363 (52%)	174 (59%)			363 (52%)	378 (56%)			174 (59%)	378 (56%)		
High	329 (48%)	123 (41%)			329 (48%)	294 (44%)			123 (41%)	294 (44%)		
M status			*0.6*	*0.8*			*<0.001*	***<0.001***			*<0.001*	***<0.001***
0	581 (84%)	256 (86%)			581 (84%)	132 (20%)			256 (86%)	132 (20%)		
1	2 (0.3%)	0 (0%)			2 (0.3%)	0 (0%)			0 (0%)	0 (0%)		
x	109 (15.7%)	41 (14%)			109 (15.7%)	540 (80%)			41 (14%)	540 (80%)		
MSI or MMR status			*0.8*	*0.8*			*>0.9*	*>0.9*			*0.8*	*>0.9*
MSS or pMMR	620 (91%)	259 (90%)			620 (91%)	520 (91%)			259 (90%)	520 (91%)		
MSI or dMMR	63 (9%)	28 (10%)			63 (9%)	53 (9.2%)			28 (10%)	53 (9.2%)		
Unknown	9	10			9	99			10	99		
Sidedness			*0.2*	*0.8*			*0.4*	*0.6*			*0.6*	*0.8*
Left	410 (59%)	163 (55%)			410 (59%)	382 (57%)			163 (55%)	382 (57%)		
Right	282 (41%)	134 (45%)			282 (41%)	290 (43%)			134 (45%)	290 (43%)		
Lung metastasis	53 (7.7%)	20 (6.7%)	*0.7*	*0.8*	53 (7.7%)	10 (6.1%)	*0.6*	*0.8*	20 (6.7%)	10 (6.1%)	*0.8*	*>0.9*
Unknown	0	0			0	509			0	509		
Liver metastasis	71 (10%)	28 (9.4%)	*0.7*	*0.8*	71 (10%)	10 (6.1%)	*0.14*	*0.4*	28 (9.4%)	10 (6.1%)	*0.3*	*0.6*
Unknown	0	0			0	509			0	509		
Bone metastasis	6 (0.9%)	1 (0.3%)	*0.7*	*0.8*	6 (0.9%)	1 (0.6%)	*>0.9*	*>0.9*	1 (0.3%)	1 (0.6%)	*>0.9*	*>0.9*
Unknown	0	0			0	509			0	509		
Other metastasis	35 (5.1%)	13 (4.4%)	*0.7*	*0.8*	35 (5.1%)	11 (6.7%)	*0.4*	*0.6*	13 (4.4%)	11 (6.7%)	*0.3*	*0.6*
Unknown	0	0			0	509			0	509		
KRAS			*0.7*	*0.8*			*0.3*	*0.6*			*0.5*	*0.8*
Mutated	321 (46%)	134 (45%)			321 (46%)	50 (41%)			134 (45%)	50 (41%)		
Wild-Type	371 (54%)	163 (55%)			371 (54%)	72 (59%)			163 (55%)	72 (59%)		
Unknown	0	0			0	550			0	550		
BRAF			*0.4*	*0.8*			*0.4*	*0.6*			*0.2*	*0.6*
Mutated	75 (11%)	37 (13%)			75 (11%)	10 (8.3%)			37 (13%)	10 (8.3%)		
Wild-Type	598 (89%)	250 (87%)			598 (89%)	111 (92%)			250 (87%)	111 (92%)		
Unknown	19	10			19	551			10	551		

MSI: Microsatellite Instability; MMR: Mismatch Repair; MSS: Microsatellite Stable; dMMR: deficient Mismatch Repair; pMMR: proficient Mismatch repair

Supplementary Table 4: Comparisons of clinical variables according to CD3 status (High vs Other) in each cohort.

Variables	Training cohort				Internal validation cohort				External validation cohort			
	High *N=283*	Other *N=409*	P-value	Adjusted p-value	High *N=118*	Other *N=179*	P-value	Adjusted p-value	High *N=262*	Other *N=410*	P-value	Adjusted p-value
Sex			0.4	0.4			0.2	0.6			0.8	>0.9
Male	165 (58%)	251 (61%)			58 (49%)	102 (57%)			153 (58%)	236 (58%)		
Female	118 (42%)	158 (39%)			60 (51%)	77 (43%)			109 (42%)	174 (42%)		
Age	60 (54, 67)	61 (54, 67)	0.2	0.3	61 (54, 67)	62 (54, 68)	0.5	0.7	64 (56, 73)	66 (58, 73)	0.3	0.6
≤60	150 (53%)	191 (47%)	0.10	0.2	56 (47%)	79 (44%)	0.6	0.7	94 (36%)	129 (31%)	0.2	0.6
>60	133 (47%)	218 (53%)			62 (53%)	100 (56%)			168 (64%)	281 (69%)		
Histopathological grade			0.4	0.4			0.2	0.6			0.5	0.8
Well/Moderately differentiated	235 (83%)	329 (80%)			102 (86%)	145 (81%)			227 (87%)	362 (88%)		
Poorly differentiated	48 (17%)	80 (20%)			16 (14%)	34 (19%)			35 (13%)	48 (12%)		
T status			0.022	0.12			0.3	0.6			0.3	0.6
1-3	238 (84%)	315 (77%)			100 (85%)	143 (80%)			206 (79%)	337 (82%)		
4	45 (16%)	94 (23%)			18 (15%)	36 (20%)			56 (21%)	73 (18%)		
N status			0.2	0.3			0.3	0.6			0.030	0.5
1	188 (66%)	253 (62%)			84 (71%)	117 (65%)			191 (73%)	266 (65%)		
2	95 (34%)	156 (38%)			34 (29%)	62 (35%)			71 (27%)	144 (35%)		
Risk Stage			0.052	0.2			0.10	0.5			0.8	>0.9
Low	161 (57%)	202 (49%)			76 (64%)	98 (55%)			149 (57%)	229 (56%)		
High	122 (43%)	207 (51%)			42 (36%)	81 (45%)			113 (43%)	181 (44%)		
M status			0.092	0.2			0.3	0.6			0.6	0.9
0	230 (81%)	351 (86%)			105 (89%)	151 (84%)			54 (21%)	78 (19%)		
1	0 (0%)	2 (0.5%)			0	0			0	0		
x	53 (19%)	56 (14%)			13 (11%)	28 (16%)			208 (79%)	332 (81%)		
MSI or MMR status			0.002	**0.016**			0.4	0.6			0.14	0.6
MSS or pMMR	240 (87%)	380 (94%)			99 (88%)	160 (91%)			192 (88%)	328 (92%)		
MSI or dMMR	37 (13%)	26 (6.4%)			13 (12%)	15 (9%)			25 (12%)	28 (7.9%)		
Unknown	6	3			6	4			45	54		
Sidedness			<0.001	**0.010**			0.020	0.3			0.3	0.7
Left	146 (52%)	264 (65%)			55 (47%)	108 (60%)			143 (55%)	239 (58%)		
Right	137 (48%)	145 (35%)			63 (53%)	71 (40%)			119 (45%)	171 (42%)		
Lung metastasis	19 (6.7%)	34 (8.3%)	0.4	0.4	7 (5.9%)	13 (7.3%)	0.7	0.7	7 (10%)	3 (3.1%)	0.093	0.5
Unknown	0	0			0	0			195	314		
Liver metastasis	21 (7.4%)	50 (12%)	0.041	0.2	7 (5.9%)	21 (12%)	0.094	0.5	3 (4.5%)	7 (7.3%)	0.5	0.8
Unknown	0	0			0	0			195	314		
Bone metastasis	1 (0.4%)	5 (1.2%)	0.4	0.4	0 (0%)	1 (0.6%)	>0.9	>0.9	0 (0%)	1 (1.0%)	>0.9	>0.9
Unknown	0	0			0	0			195	314		
Other metastasis	10 (3.5%)	25 (6.1%)	0.13	0.2	4 (3.4%)	9 (5.0%)	0.5	0.7	4 (6.0%)	7 (7.3%)	>0.9	>0.9
Unknown	0	0			0	0			195	314		
KRAS			0.2	0.2			>0.9	>0.9			0.067	0.5
Mutated	122 (43%)	199 (49%)			53 (45%)	81 (45%)			16 (31%)	34 (48%)		
Wild-Type	161 (57%)	210 (51%)			65 (55%)	98 (55%)			35 (69%)	37 (52%)		
Unknown	0	0			0	0			211	339		
BRAF			0.10	0.2			0.3	0.6			0.7	>0.9
Mutated	37 (14%)	38 (10%)			12 (11%)	25 (14%)			5 (10%)	5 (7.0%)		
Wild-Type	236 (86%)	362 (90%)			102 (89%)	148 (86%)			45 (90%)	66 (93%)		
Unknown	10	9			4	6			212	339		

MSI: Microsatellite Instability; MMR: Mismatch Repair; MSS: Microsatellite Stable; dMMR: deficient Mismatch Repair; pMMR: proficient Mismatch Repair

Supplementary Table 5: Comparisons of clinical variables according to CD3ML status in each cohort.

Variables	Training cohort				Internal validation cohort				External validation cohort			
	Low N=478	High N=214	P-value	Adjusted p-value	Low N=157	High N=140	P-value	Adjusted p-value	Low N=373	High N=299	P-value	Adjusted p-value
Sex			0.2	0.3			0.9	>0.9			0.4	0.5
Male	279 (58%)	137 (64%)			84 (54%)	76 (54%)			210 (56%)	179 (60%)		
Female	199 (42%)	77 (36%)			73 (46%)	64 (46%)			163 (44%)	120 (40%)		
Age	60 (53, 67)	62 (55, 67)	0.11	0.3	62 (53, 68)	61 (55, 67)	>0.9	>0.9	64 (57, 72)	67 (58, 75)	**0.004**	**0.036**
≤60	241 (50%)	100 (47%)	0.4	0.5	69 (44%)	66 (47%)	0.6	>0.9	136 (36%)	87 (29%)	0.044	0.2
>60	237 (50%)	114 (53%)			88 (56%)	74 (53%)			237 (64%)	212 (71%)		
Histopathological grade			0.3	0.4			0.046	0.11			0.8	>0.9
Well/Moderately differentiated	395 (83%)	169 (79%)			137 (87%)	110 (79%)			326 (87%)	263 (88%)		
Poorly differentiated	83 (17%)	45 (21%)			20 (13%)	30 (21%)			47 (13%)	36 (12%)		
T status			0.014	0.055			0.049	0.11			0.13	0.3
1-3	394 (82%)	159 (74%)			135 (86%)	108 (77%)			309 (83%)	234 (78%)		
4	84 (18%)	55 (26%)			22 (14%)	32 (23%)			64 (17%)	65 (22%)		
N status			**<0.001**	**0.002**			**<0.001**	**<0.001**			0.2	0.4
1	326 (68%)	115 (54%)			122 (78%)	79 (56%)			261 (70%)	196 (66%)		
2	152 (32%)	99 (46%)			35 (22%)	61 (44%)			112 (30%)	103 (34%)		
Risk Stage			**<0.001**	**0.001**			**<0.001**	**<0.001**			0.080	0.2
Low	275 (58%)	88 (41%)			109 (69%)	65 (46%)			221 (59%)	157 (53%)		
High	203 (42%)	126 (59%)			48 (31%)	75 (54%)			152 (41%)	142 (47%)		
M status			0.5	0.5			0.8	>0.9			**0.004**	**0.036**
0	398 (83%)	183 (86%)			136 (87%)	120 (86%)			88 (24%)	44 (15%)		
1	1 (0.2%)	1 (0.5%)			0	0			0	0		
x	79 (17%)	30 (14%)			21 (13%)	20 (14%)			285 (76%)	255 (85%)		
MSI or MMR status			0.029	0.093			0.10	0.2			0.9	>0.9
MSS or pMMR	419 (89%)	201 (94%)			133 (88%)	126 (93%)			298 (91%)	222 (91%)		
MSI or dMMR	51 (11%)	12 (5.6%)			19 (12%)	9 (6.7%)			31 (9.4%)	22 (9.0%)		
Unknown	8	1			5	5			44	55		
Sidedness			0.4	0.5			0.6	>0.9			0.086	0.2
Left	278 (58%)	132 (62%)			84 (54%)	79 (56%)			223 (60%)	159 (53%)		
Right	200 (42%)	82 (38%)			73 (46%)	61 (44%)			150 (40%)	140 (47%)		
Lung metastasis	34 (7.1%)	19 (8.9%)	0.4	0.5	6 (3.8%)	14 (10%)	0.034	0.11	7 (6.6%)	3 (5.3%)	>0.9	>0.9
Unknown	0	0			0	0			267	242		
Liver metastasis	44 (9.2%)	27 (13%)	0.2	0.3	9 (5.7%)	19 (14%)	0.021	0.11	6 (5.7%)	4 (7.0%)	0.7	>0.9
Unknown	0	0			0	0			267	242		
Bone metastasis	4 (0.8%)	2 (0.9%)	>0.9	>0.9	1 (0.6%)	0 (0%)	>0.9	>0.9	0 (0%)	1 (1.8%)	0.3	0.5
Unknown	0	0			0	0			267	242		
Other metastasis	16 (3.3%)	19 (8.9%)	**0.002**	**0.011**	3 (1.9%)	10 (7.1%)	0.028	0.11	3 (2.8%)	8 (14%)	0.017	0.091
Unknown	0	0			0	0			267	242		
KRAS			>0.9	>0.9			0.7	>0.9			0.087	0.2
Mutated	222 (46%)	99 (46%)			69 (44%)	65 (46%)			31 (36%)	19 (53%)		
Wild-Type	256 (54%)	115 (54%)			88 (56%)	75 (54%)			55 (64%)	17 (47%)		
Unknown	0	0			0	0			287	263		
BRAF			0.3	0.5			0.9	>0.9			0.2	0.3
Mutated	48 (10%)	27 (13%)			20 (13%)	17 (12%)			5 (5.9%)	5 (14%)		
Wild-Type	415 (90%)	183 (87%)			131 (87%)	119 (88%)			80 (94%)	31 (86%)		
Unknown	15	4			6	4			288	263		

MSI: Microsatellite Instability; MMR: Mismatch Repair; MSS: Microsatellite Stable; dMMR: deficient Mismatch Repair; pMMR: proficient Mismatch repair

Supplementary Figure 1: Flowcharts of study

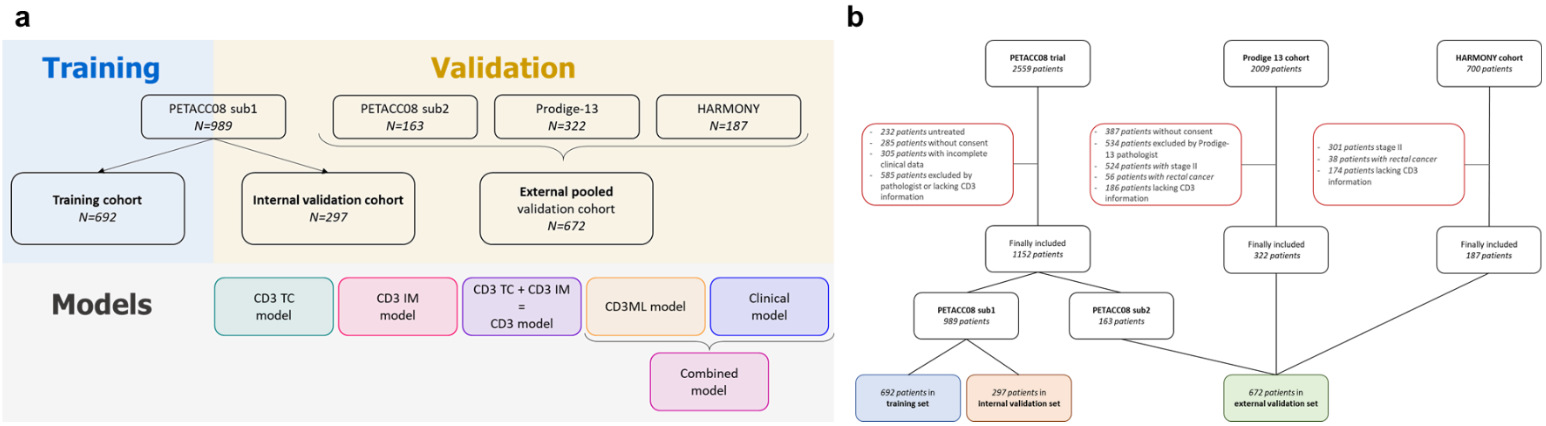

a) Flowchart of study cohorts and models. b) Consort diagram of patients included or excluded for study.

Supplementary Figure 2: Clinical variables and digital scores

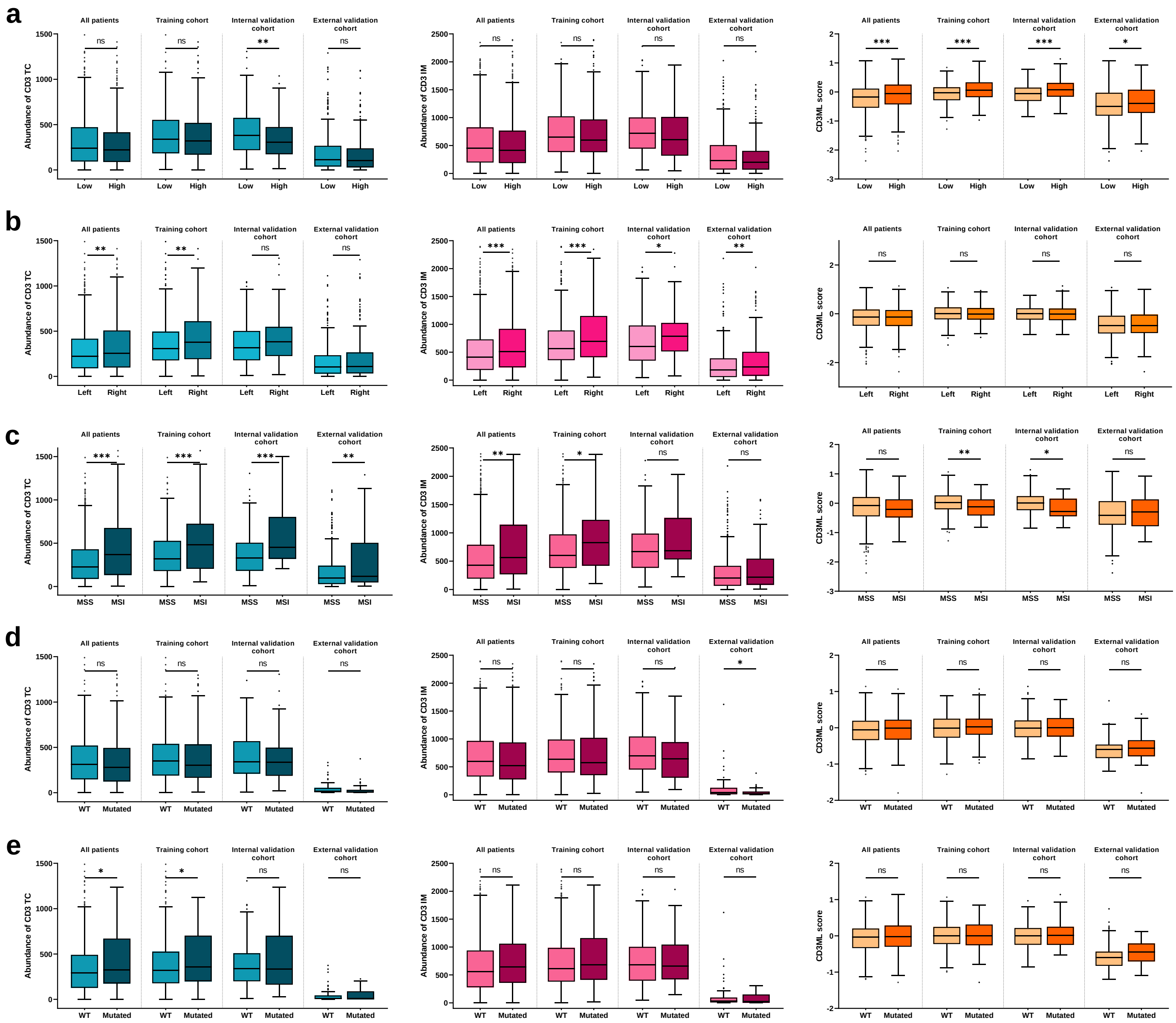

Boxplots showing CD3 TC (blue), CD3 IM (pink) and CD3ML (orange) score expression for the whole cohort and for the training, internal validation and external validation cohorts according to a) risk stage, b) sidedness, c) MSI/MMR status, d) KRAS mutated status and e) BRAF mutated status.

Wilcoxon tests: *p<0·05, **p<0·01 and ***p<0·001

TC: Tumour Core; IM: Invasive Margin; MSI: Microsatellite Instability; MMR: Mismatch repair

Supplementary Figure 3: Association between CD3 and CD3ML scores

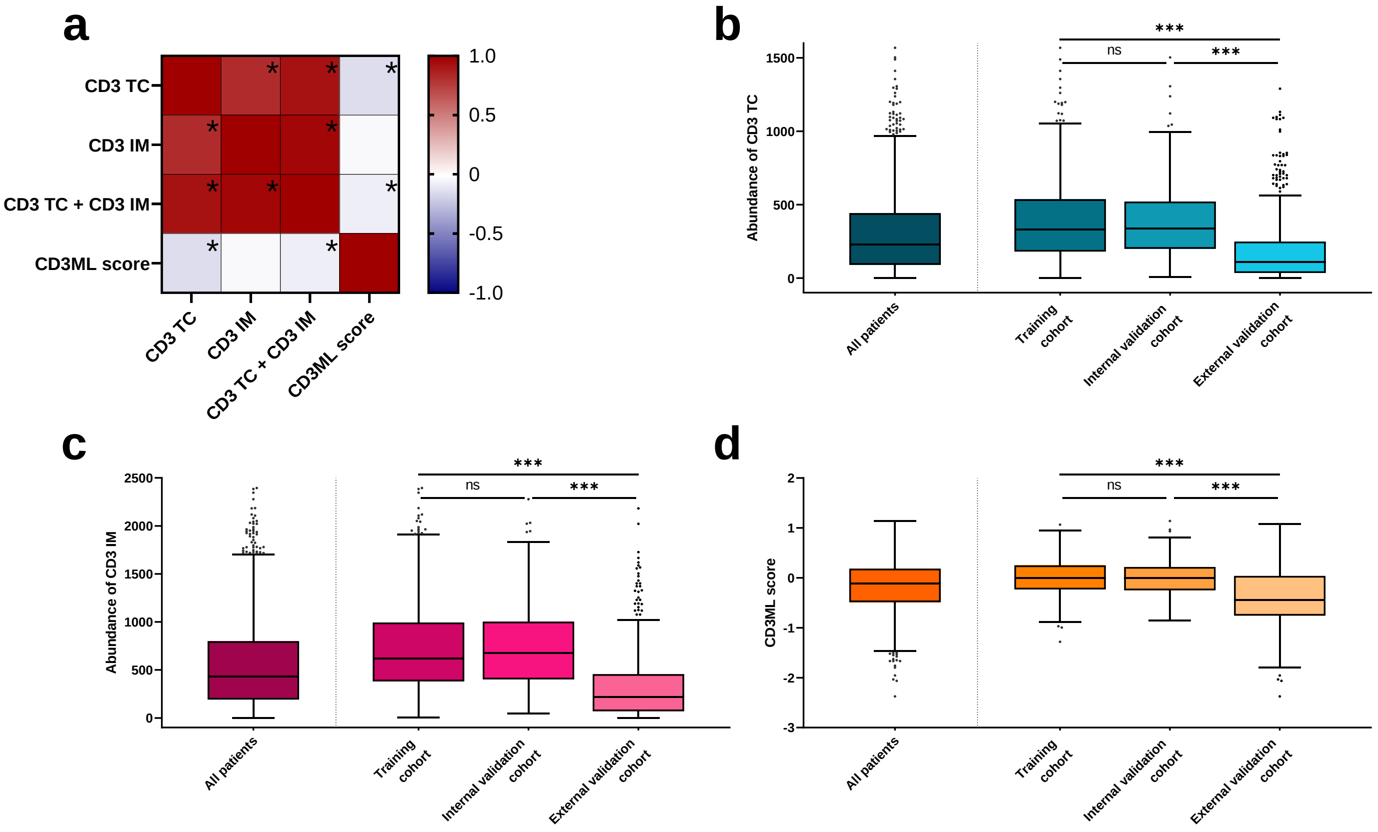

a) Heatmap showing the correlation matrix between CD3 TC, IM, CD3TC+CD3IM and CD3ML scores; correlations were calculated with Pearson's correlation coefficient and *p-value<0.05* are represented by a star.
Boxplots showing b) CD3 TC score, c) CD3 IM score and d) CD3ML score in all patients according to training, internal validation and external validation cohorts.
Wilcoxon tests: **p*<0·05, ***p*<0·01 and ****p*<0·001
TC: Tumour Core; IM: Invasive Margin

Supplementary Figure 4: Association between CD3ML score and N status in all cohort.

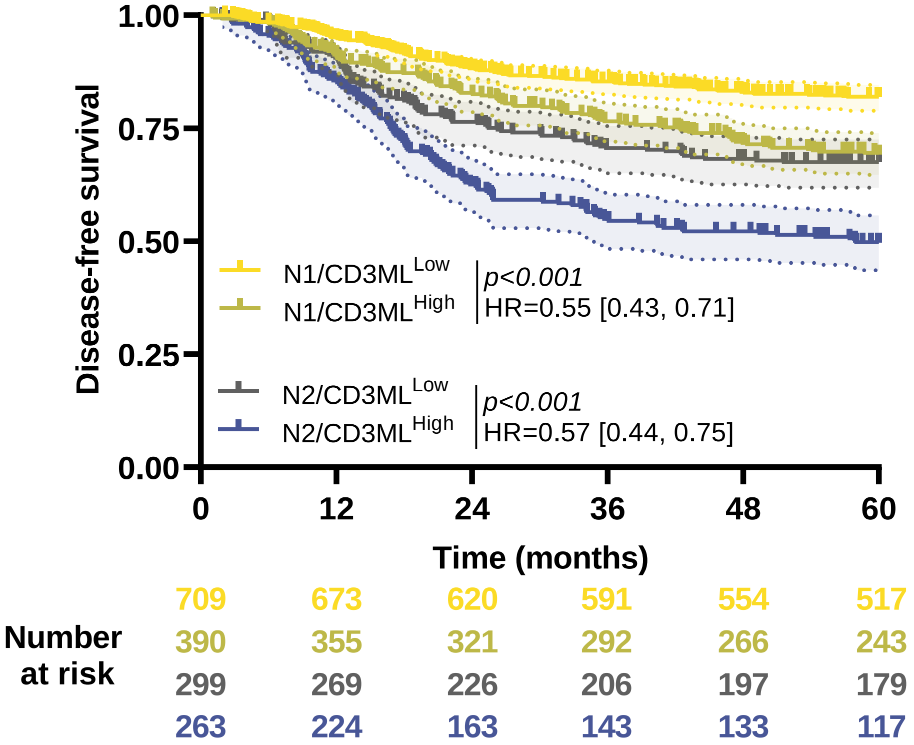

Kaplan-Meier curves with patients stratified according to CD3ML and N status for disease-free survival in all patients. Hazard ratio and log-rank test p-value were shown when proportional hazards assumption was verified, otherwise the RMST measure and p-value were shown.

Supplementary Figure 5: Association between risk stage and CD3ML score in each cohort

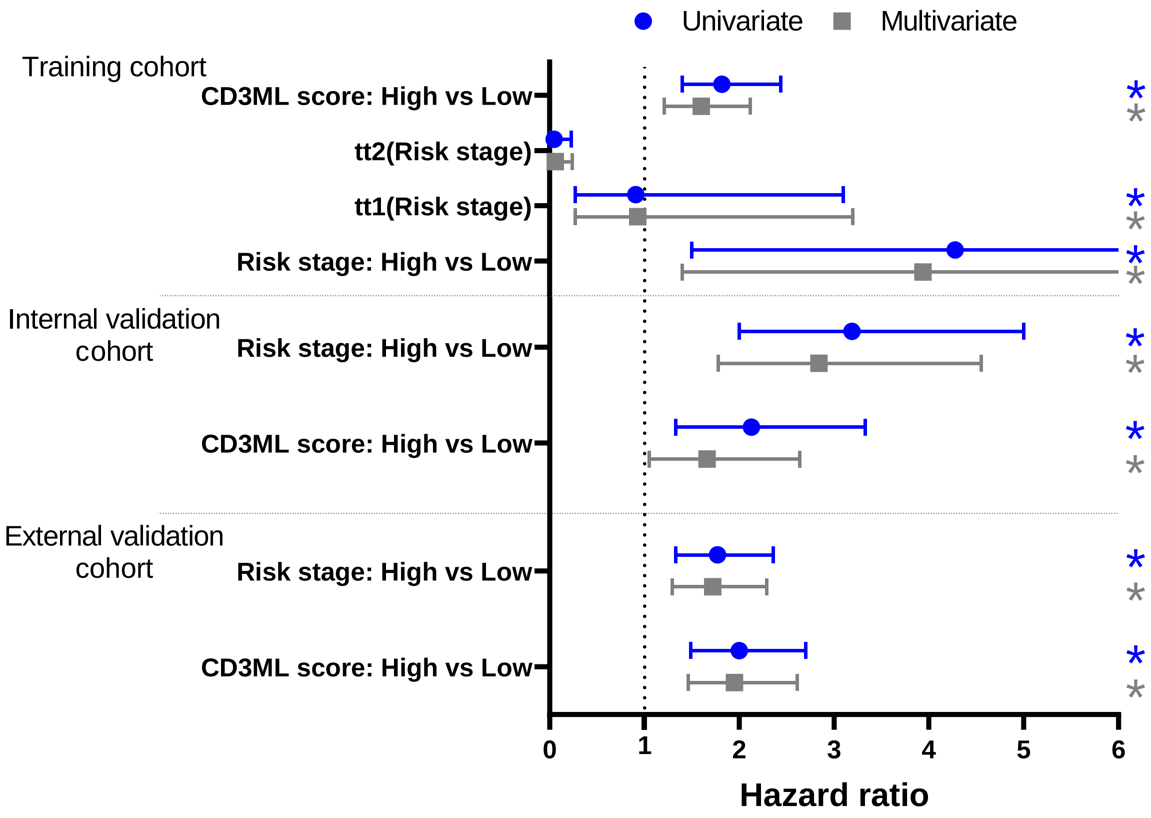

Forest plots representing hazard ratios and confidence intervals for univariate (blue) and multivariate (grey). Cox models for disease-free survival estimated using risk stage and CD3ML score in each cohort. *: Log-rank test p-value≤0·05. Risk stage variable was estimated using time-dependent coefficient. Coefficients for tt1(Risk stage) and tt2(Risk stage) describe time varying effect of risk stage evaluated between 0 and 22 months, and 22 and 60 months, respectively.

Supplementary Figure 6: Predictive power of clinical and digital variables

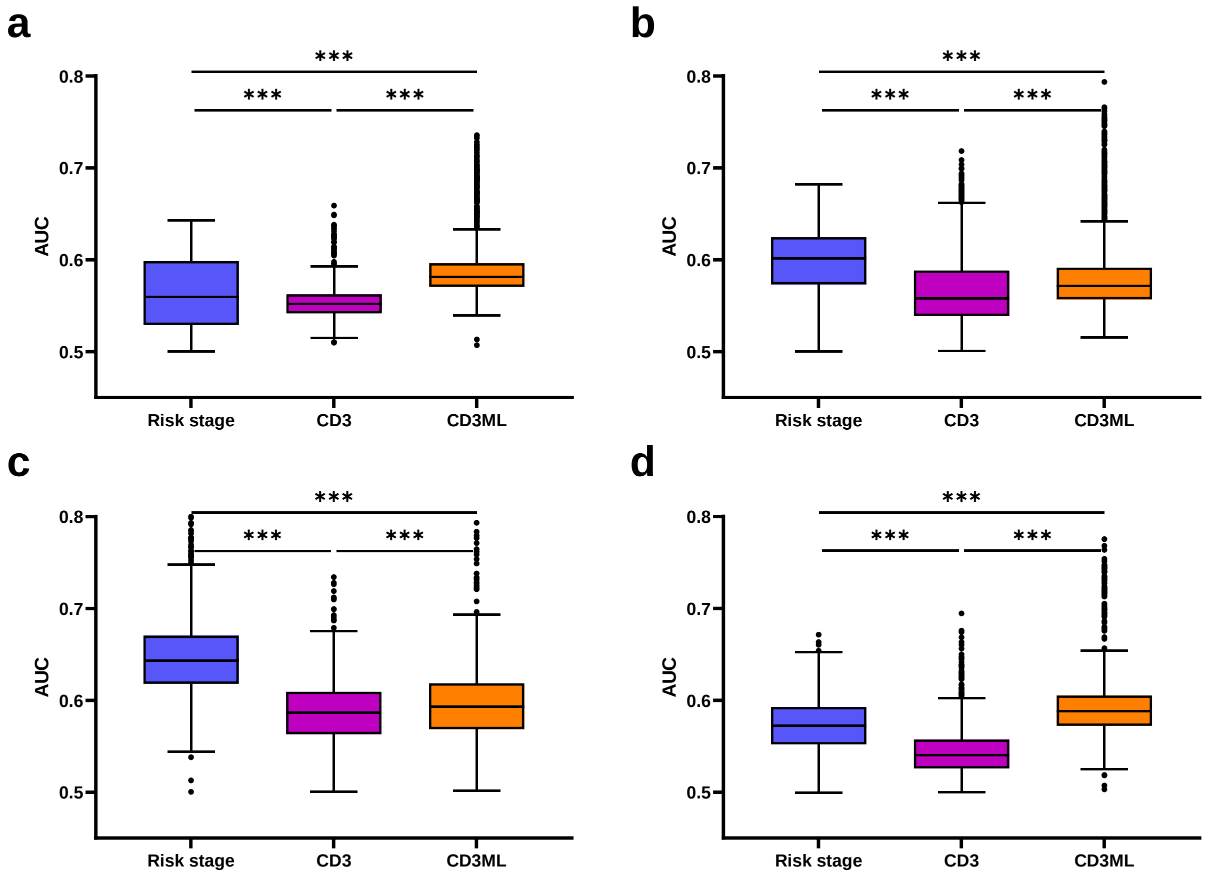

Boxplots showing the area under the curve (AUC) of different variables of interest using a 1000x bootstrap strategy in a) the whole cohort, b) the training cohort, c) the internal validation cohort and d) the external validation cohort. Wilcoxon tests: ****p*<0·001
